# 4527 Assessing Quality of Life, Depression, and Symptomatology in Puerto Rican Hispanic Patients Hospitalized with Heart Failure

**DOI:** 10.1017/cts.2020.246

**Published:** 2020-07-29

**Authors:** Ariel Gonzalez-Cordero

**Affiliations:** 1 University of Puerto Rico-Medical Sciences Campus

## Abstract

OBJECTIVES/GOALS: Heart failure is a public health problem. Currently, heart failure affects 2-5 % of adults within the age of 65-75 years. (Mosterd & Hoes, 2007)Moreover, rates of hospitalization and rehospitalization among patients with heart failure are high and are associated with poor quality of life(Dunlay et al., 2011)Unsurprisingly, studies have found that poor quality of life is linked to decreased physical activity and increased symptomatology, a perception that can quickly change depending on the patient’s mood. Factors such as age, cultural background, socioeconomical status, ethnicity, and gender are highly correlated with quality of life but have not been studied thoroughly. Quality of life assessment in Puerto Rican Hispanics living with heart failure is non-existent. Objective:•To determine gender-specific differences in quality of life for patients hospitalized due to heart failure in Puerto Rico.•To correlate heart failure symptoms, presence of depression and level of perceived quality of life in Puerto Rican patients hospitalized due to heart failure METHODS/STUDY POPULATION: We will recruit patients admitted with heart failure (n = 300) to the Cardiovascular Hospital of Puerto Rico and The Caribbean between 2019-2021. In the first aim, we will implement the Minnesota Living with Heart Failure Questionnaire to assess the quality of life of Puerto Rican Hispanics diagnosed that life with heart failure and the short form-36 (SF-36) for a generic quality of life assessment. For the second aim, we will provide two instruments: The Geriatrics DepressionScale QuestionnaireShort Form (GDS-SF)and the Memorial Symptom Assessment ScaleShort Form (MSAS-SF) to assess the presence and severity of depression and multiple general symptoms RESULTS/ANTICIPATED RESULTS: We expect that women living with heart failure will have worse quality of life and higher NYHA scale and NT-pro-BNP. DISCUSSION/SIGNIFICANCE OF IMPACT: This contribution is significant because it can clarify the specific risk factors in the Puerto Rican community that are associated with lower quality of life among patients suffering from heart failure. This, in term, can allow physicians to identify which population of HF patients is at risk,and have strategies to improve quality of life

